# Avian metapneumovirus: A five-plex digital droplet RT-PCR method for identification of subgroups A, B, C, and D

**DOI:** 10.3389/fvets.2022.1058294

**Published:** 2022-11-15

**Authors:** Evelyne Lemaitre, Stéphanie Bougeard, Chantal Allée, Nicolas Eterradossi, Céline Courtillon, Paul Alun Brown

**Affiliations:** ^1^Ploufragan-Plouzané-Niort Laboratory, French Agency for Food, Environmental and Occupational Health & Safety (Anses), VIPAC Unit, WOAH Reference Laboratory for Avian Metapneumovirus Infections, Ploufragan, France; ^2^Ploufragan-Plouzané-Niort Laboratory, French Agency for Food, Environmental and Occupational Health & Safety (Anses), EPISABE Unit, Ploufragan, France

**Keywords:** avian metapneumovirus, digital droplet, identification, five-plex, method

## Abstract

End-point and real-time avian metapneumovirus (AMPV) RT-PCRs have been developed to detect one or two of the four recognized subgroups (A,B,C, and D) simultaneously or for broad range AMPV detection. Current subgroup specific tests target variable areas of the genome which makes these PCRs sensitive to specificity defects as recently documented. In the current study, a single five-plex digital droplet RT-PCR targeting the conserved viral polymerase gene of AMPV, which is less prone to genetic drift, has been designed. This digital droplet RT-PCR was capable of identifying each of the four AMPV subgroups. Each subgroup was identified according to a specifically assigned fluorescent amplitude. Specificity, which was tested including 31 AMPV strains, non-AMPV avian viruses and closely related human respiratory viruses, was 100%. The specific limit of detection for extracted viral RNA was estimated between 1 and 3 copies/μl. This tool simplifies the number of tests required for AMPV genotype diagnostics and should be theoretically less effected by viral genome evolution due to its target region. Ultimately, application of this test will contribute to an improved understanding of the global geographic distribution and subgroup host range of field strains.

## Introduction

Since their first detection in South Africa in the late 1970s, avian metapneumoviruses (AMPV), family *Pneumoviridae*, genus *Metapneumovirus* (1), have now been detected in most regions of the world (2). AMPV fundamentally cause respiratory and reproductive diseases in poultry and have had serious economic impact on the poultry industry. To date, based on genetic and antigenic differences, four subgroups are described (AMPV-A, B, C, and D) (3–8) and two genetic lineages of AMPV-C (8) have been identified. Recently, two divergent AMPVs from a monk parakeet (9) and a gull (10) were described and do not cluster genetically with any of the four previously described subgroups. The principal host species for AMPVs are turkeys, chickens and ducks, however, they have also been isolated from pheasants (11), and been detected in guinea fowls (12), or wild water fowls (13) by serological and molecular methods, respectively.

Concerning field detection of AMPVs in the three principal poultry species (turkeys, chickens and ducks), all subgroups have been detected in turkeys (14–17), although to date, the subgroup C viruses detected in this species have been of a lineage unique to North America (US AMPV-C). In domestic Muscovy and Pekin ducks, only AMPV-Cs of a Eurasian lineage have been reported (18, 19). In chickens, AMPV A, B and an AMPV-C have been described (20–24), however the lineage of the latter is yet to be defined. Recently, details on the extent of infection of the four recognized subgroups and lineages of AMPV in turkeys, chickens and Muscovy ducks under controlled experimental conditions have been published (25). Interestingly, apart from confirming the subgroup / host relationships described above, this study showed that AMPV-C viruses of both US and Eurasian lineage could be re-isolated from chickens, and AMPV-C viruses of Eurasian lineage from turkeys despite a lack of viral RNA detection using a well-characterized real-time RT-PCR (26) targeting the hypervariable SH gene. These results demonstrate that replication of AMPVs in a “non-conventional host” may modify the viral consensus sequence, especially in hypervariable regions of the genome, and thus go undetected by molecular tests, such as those currently available, that target these regions.

To date, both classical and real-time RT-PCRs have been developed for either broad range (3, 27) or subgroup specific detection. Concerning real-time RT-PCR tests that are subgroup specific, tests exist in duplex for subgroups A and B (26, 28, 29), in triplex for subgroup C (both lineages) and the new identified parakeet and gull AMPVs (30) and in individual tests for subgroups C and D (26). All these current subgroup specific molecular tests are based in the hypervariable G or SH genes except for that detecting the AMPV gull virus which is in the polymerase L gene.

In this context, the development of a multiplex reaction PCR fully based on a non-hypervariable region of the genome that is capable of detecting and differentiating the four recognized subgroups found in poultry would be beneficial. Theoretically, this could be addressed using recent real-time PCR machines that now have the capability of reading up to four different fluorescent wavelengths. In reality however, real-time PCRs that differentiate more than three targets are rare due to the level of competition between primers, probes and target molecules within the reaction mixture. The technology of droplet digital PCR (ddPCR), based on the amplification of target molecules of the same mixture but distributed into thousands of reaction droplets, reduces this competition and results in an improved capacity for multiplexing (31). ddPCR is based on end-point PCR reactions and uses droplet reader machines capable of reading several fluorescent wave lengths. Different positive targets can be separated into specific clusters using probes labeled with different fluorescent dyes but also by using probes at different concentrations that are labeled with the same dye. The method of generating specific clusters by varying probe concentrations is based on the fact that the fluorescent amplitude value is directly linked to the number of probe molecules in the PCR reaction mixture. Combining these two techniques means that many specific clusters can be obtained even with a two-color reader.

The current study describes the development of a five-plex RT-ddPCR for the identification of AMPV-A, B, C or D and the housekeeping gene Glyceraldehyde-3-phosphate dehydrogenase (GAPDH). For the amplification of AMPV molecules, two common primers and four subgroup specific probes were used which targeted a small region of the conserved viral polymerase gene. Distinction between subgroups was achieved on the principle of varying probe concentrations.

## Materials and methods

### Ethic statements

Tracheal swab samples were taken during previous animal experiments performed at Anses Ploufragan (25). These animal experiments were approved by Anses Ploufragan ethical committee (committee number C2EA-016/ ComEth ANSES/ENVA/UPEC) and authorized by French Ministry for higher education and research (application number 14-024, statement number 08/04/14-7). Chickens were raised and humanely euthanized in agreement with EU directive number 2010/63/UE.

### Viruses

Twenty-eight AMPV field strains that had been isolated on Vero cells as previously described (32), three AMPV vaccine strains, 35 AMPV positive tracheal swab samples from previously performed experimental trials in birds (27) and 35 non-avian metapneumovirus strains were used as test samples. The subgroup assignment of AMPVs had previously been determined using existing well-characterized diagnostic methods (26) and Sanger sequencing. A reference virus of each subgroup was also tested (AMPV-A 85051, AMPV-B 86004, AMPV-C 99178, and AMPV-D 85035) for which the titers had been previously determined using the method of Reed and Muench (33) to be 10^5.2^, 10^5.9^, 10^6.1^, and 10^5.6^ TCID_50_/ml, respectively.

### Viral RNA extraction

Viral RNA was extracted from all of the above samples as described previously (27) using QIAamp Viral RNA Mini Kit (Qiagen, France, reference 52906).

### Primer design

Based on a nucleotide alignment using MEGA software version 7 (34) of all available AMPV full length sequences on the database, a zone in the L gene (position 1980–2140) that allowed for the design of one single forward and one single reverse primer that were common to all subgroups and for the design of subgroups specific probes was selected (Table 1). Primers and probes were evaluated *in silico* using OligoAnalyzer Tool (https://eu.idtdna.com/pages/tools/oligoanalyzer) for secondary structures and self- and hetero-dimers. Specificity was investigated using Primer-BLAST tool. Probes were double-quenched with 3′ Iowa Black FQ quencher and an internal ZEN quencher to decrease the background. Probes for AMPV-A, B and D used 5′ 6-carboxyfluorescein (FAM) and probes for AMPV-C and an endogenous reference gene, the house keeping gene GAPDH (Table 1) used 5′ hexachloro-fluorescein phosphoramidite (HEX).

**Table 1:** Details of the five-plex RT-ddPCR AMPV primers and probes.

**Name**	**Target**	**Sequence 5′-3′**	**L ORF position**	**Amplicon size**
PanAMPV L1Fwd	AMPV-A, B, C, and D	CACAGAGTCTATTYTGCTGG	1,980–2,001^c^	177 pb
PanAMPV L1Rev	AMPV-A, B, C, and D	TCCACATCTTTTGRCACCA	2,140–2,158^c^	
AMPVA L1Pro	AMPV A	FAM-TCTGGTCTA-ZEN-TGTCGTAGATTCCACC-IABkFQ	2,065–2,089^a^	
AMPVB L1Pro	AMPV B	FAM-CTATACAAC-ZEN-CCACTCTGCTCAGGGATTGA-IABkFQ	2,086–2,114^b^	
AMPVC L1Pro	AMPV C	HEX-AGACATGCC-ZEN-CCTCCAGAAACAGAAGGAGT- IABkFQ	2,044–2,072^c^	
AMPVD L1Pro	AMPV D	FAM-CTGCTCAGG-ZEN-TATGTTGTCTATGTCATATTC-IABkFQ	2,071–2,100^d^	
PanGAPDH Fwd	GAPDH	TGAGTATGTTGTGGAGTCCACT		198 pb
PanGAPDH Rev	GAPDH	GCCAGGCAGTTGGTGGTGCA		
PanGAPDH Pro	GAPDH	HEX-TGAGCCCCA-ZEN-GCCTTCTCCATGGA-IABkFQ		

a AMPV reference strain DQ666911.

b AMPV reference strain AB548428.

c AMPV reference strain HG934338.

d AMPV reference strain HG934339.

### Digital droplet RT-PCR

The RT-ddPCR platform used enables the detection of two fluorophores: FAM in channel 1 (CH1) and HEX in channel 2 (CH2). Based on implementation of varying probe concentrations, a five-plex reaction was possible by assigning AMPV-A, B and D AMPV targets to CH1 and AMPV-C and GAPDH to CH2. RT-ddPCR assays were performed in a 20 μl final volume in RNase free water using the One-step RT-ddPCR Advanced Kit for Probe (Bio-Rad, France, reference 1864022), containing 5 μl of Supermix, 2 μl of Reverse transcriptase, 1 μl of 300 mM DTT, 1 μl of RNA template and primers at final concentrations of 900 nM. For CH1, 300 mM of the AMPV-A probe, 200 mM of the AMPV-B probe, and 100 mM of the AMPV-D probe was used per reaction. For CH2, the concentration of probes was 300 mM for AMPV-C and 100 mM for GAPDH. The reaction mixture was then loaded into the well of an eight-channel disposable droplet generator cartridge and water-oil emulsified into 20,000 droplets with QX200 Droplet Generator (Bio-Rad, France). After emulsion, the droplets were transferred to a 96-well PCR plate for the RT-ddPCR reaction (C1000 Touch Thermal Cycler, Bio-Rad, France). The cycling conditions were as follows: 50°C for 60 min (RT reaction), 95°C for 10 min, followed by 40 cycles of 95°C for 30 s, 54°C for 30 sec, and 60°C for 60 s. A final cycle of 98°C for 10 min was performed followed by a hold temperature of 12°C. Reactions were read in a QX200 Droplet Reader (Bio-Rad, France). The results were analyzed by QuantaSoft software (version 1.7.4 Bio-Rad, France).

### Data analysis

**Figure 1:**
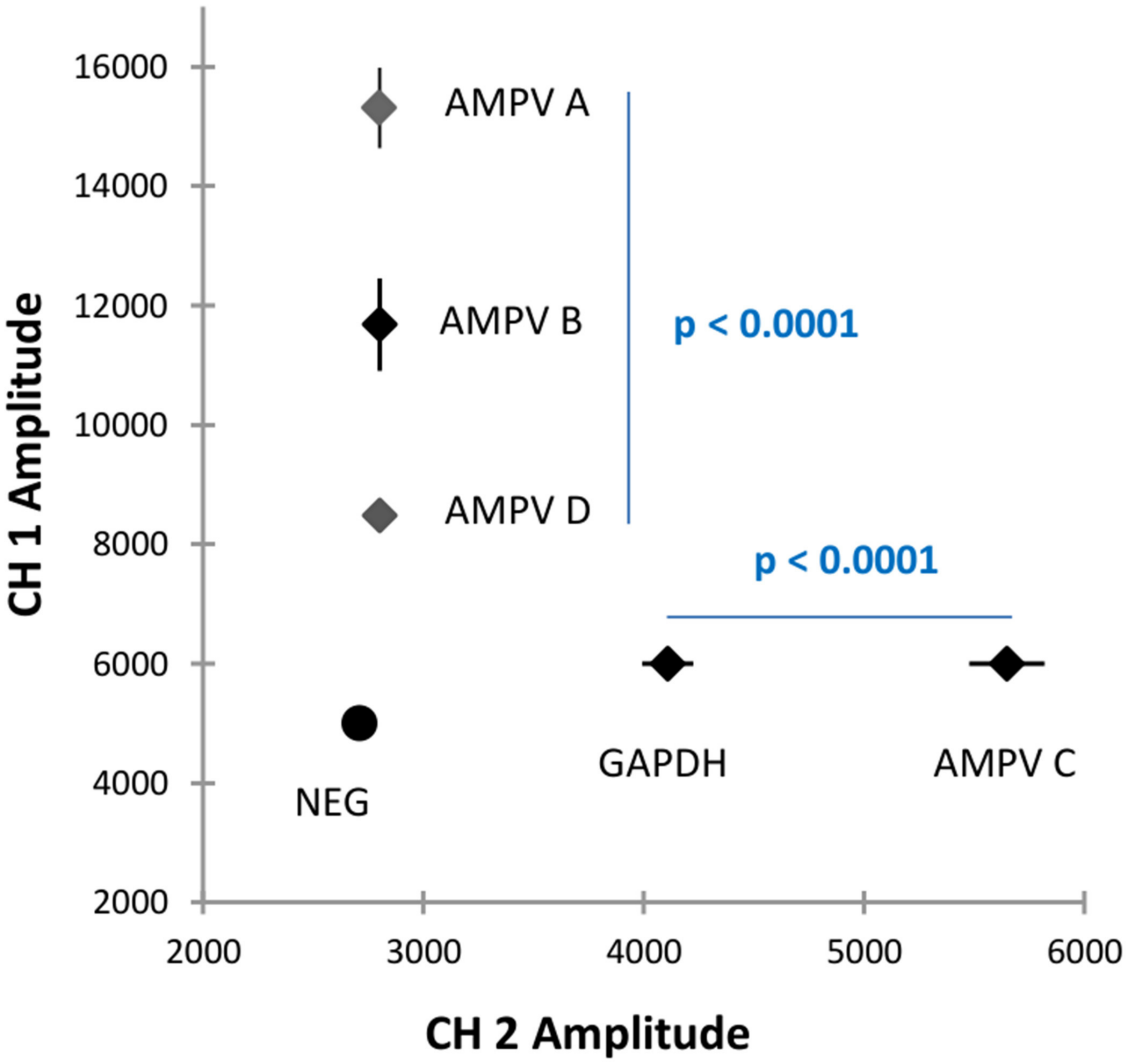
Graphical 2D plot of the five-plex RT-ddPCR showing different clusters for each AMPV subgroup, each AMPV subgroup plus GAPDH and the negative cluster.

Droplet clusters were evaluated in 2D plots as shown in Figure 1 using QuantaSoft software version 1.7.4 Bio-Rad, France). A cut-off amplitude threshold, set at ~6,000 for CH1 and 3,200 for CH2, distinguished positive droplets (one or more than one copy of the target) from negative droplets (devoid of target molecules). Ten or more positive droplets was considered as a positive result. The mean amplitude values for positive clusters in CH1 and CH2 for test samples were compared with those of AMPV reference strains to assign the AMPV subgroup. An absolute quantification of the clusters was performed.

### Probe interference test

Each probe used collectively in the five-plex RT-ddPCR was tested individually in its own simplex test. AMPV-B, C, and D were tested at 10^4^-10^2^ TCID_50_/ml and AMPV-A at 10^4^-10^1^ TCID_50_/ml. Composition of individual reactions and cycling conditions were identical to those described for the five-plex. The ratio of extracted viral RNA log_10_ copies/μl obtained by simplex RT-ddPCR compared to five-plex RT-ddPCR was evaluated. Comparison of performance of multiplex and simplex RT-PCR reactions are not documented by published guidelines but a bias above 25% has previously been reported to be significant (35). Thus, a result below 25% (absolute value) between the simplex and five-plex reactions for each subgroup would signify no interference between probes when combined into one single reaction. A Pearson's correlation coefficient was used to determine the correlation between the methods along the range.

### Specificity

The specificity of the method was evaluated by testing the 28 AMPV field strains, the three attenuated vaccine strains, the 35 AMPV-positive tracheal swabs, four human metapneumoviruses (HMPV), three respiratory syncytial virus (RSV) and various avian viruses of others families. Details of these are shown in section “Viruses” and in Table 2. For AMPV samples, the mean and standard deviation (SD) of the amplitude values in the CH1 and CH2 channels were calculated. Non-parametric Kruskal-Wallis test was applied to confirm statistical differences between the amplitude values of the different subgroups.

**Table 2:** Results of the five-plex RT-ddPCR AMPV with viruses used for specificity assessments.

**Family**	**Genus**	**Species**	**Virus strains**	**Positive/Total**
*Pneumoviridae*	*Metapneumovirus*	Avian metapneumovirus	AMPV A (T, V)	10+/10
			AMPV B (T, C, V)	12+/12
			AMPV C (T, D)	7+/7
			AMPV D (T)	2+/2
		Human metapneumovirus	HMPV A1, A2, B1, B2	0+/4
	*Orthopneumovirus*	Human respiratory virus	RSV A_long_, A2, B1	0+/3
*Orthomyxoviridae*	*Alphainfluenzavirus*	/	H1N1, H5N2, H5N3, H7N1, H7N3	0+/7
*Paramyxoviridae*	*Newcastle disease virus*	/	PMV1	0+/2
	*Avian paramyxovirus*	/	PMV2–4, 6–9, 11	0+/8
*Adenoviridae*	*Aviadenovirus*	/	Fowl adenovirus type 1	0+/1
	*Siadenovirus*	/	VEH	0+/1
*Birnaviridae*	*Avibirnavirus*	/	IBDV serotype 1,2	0+/2
*Picornaviridae*	*Tremovirus*	/	Avian encephalomyelitis virus	0+/1
*Reoviridae*	*Orthoreovirus*	/	Avian reovirus	0+/1
*Coronoviridae*	*Gamma coronavirus*	/	IBV, TCoV	0+/4
*Herpesviridae*	*Iltovirus*	Gallid alphaherpesvirus 1	ILTV	0+/1

T, turkeys; C, chickens; D, ducks; V, vaccine.

### Sensitivity and linearity

A dilution series was prepared for AMPV reference viruses by diluting viral suspension in a supernatant of specific pathogen-free (SPF) turkey tracheal swabs. Five replicates of the dilution series were performed (two independent assays, three repeats for the first assay and two repeats for the second). The determination of the limit of detection (LoD) and the limit of quantification (LoQ) were based on these samples. The LoD was the last dilution in which all replicates were positive and the LoQ was the last dilution in which all replicates showed a coefficient of variation (CV) in inter-assays of less than 25%. In ddPCR, the absolute quantification can be estimated from the number of positive droplets to the total number of droplets and their known volume with an optimal precision of concentration at 1.6 molecules per droplet.

To avoid saturation of positive droplets by target viral RNA molecules, a range from 10^4^ to 10^1^ TCID_50_/ml was selected to evaluate the linearity of the method. 2D plots from absolute quantification (log_10_ viral RNA copies/μl) against the viral titer (log_10_ TCID_50_/ml) for the four reference viruses were generated and the coefficient of determination (*R*^2^) were calculated for each subgroup.

## Results

### Probe interference test

Assays were done to compare the performance of probes in simplex and five-plex reactions by determining the absolute quantification of extracted viral RNA (log_10_ copies/μl) from reference viruses of each subgroup. All biases between the two methods were ≤25% (absolute values) except for AMPV-D at a viral titer of 10^2^ TCID_50_/ml (Table 3). To determine the correlation of the two methods, a Person's correlation coefficient for dynamic range was calculated and was equal to 0.995 (*p*-value <0.0001). Thus, significant correlation was observed between the simplex and the five-plex tests.

**Table 3:** Comparison between five-plex and simplex RT-ddPCR.

**Viral titer^a^**	**AMPV-A**			**AMPV-B**			**AMPV-C**			**AMPV-D**		
	**Five-plex^b^**	**Simplex^b^**	**Bias**	**Five-plex^b^**	**Simplex^b^**	**Bias**	**Five-plex^b^**	**Simplex^b^**	**Bias**	**Five-plex^b^**	**Simplex^b^**	**Bias**
4	3.27	3.46	5.49	2.87	3.10	7.42	2.79	2.85	2.11	2.57	2.90	11.38
3	2.27	2.48	8.47	1.84	2.03	9.36	1.86	1.89	1.59	1.60	1.89	15.34
2	1.24	1.46	15.07	0.91	1.12	18.75	0.89	0.85	−4.71	0.61	0.92	33.70
1	0.44	0.41	−7.32									

a Log_10_ (TCID_50_/ml).

b Log_10_ (absolute copies/μl of extracted viral RNA), Bias: expressed in % [calculation: 100–(five-plex result/Simplex result^*^100)].

### Specificity

**Figure 2:**
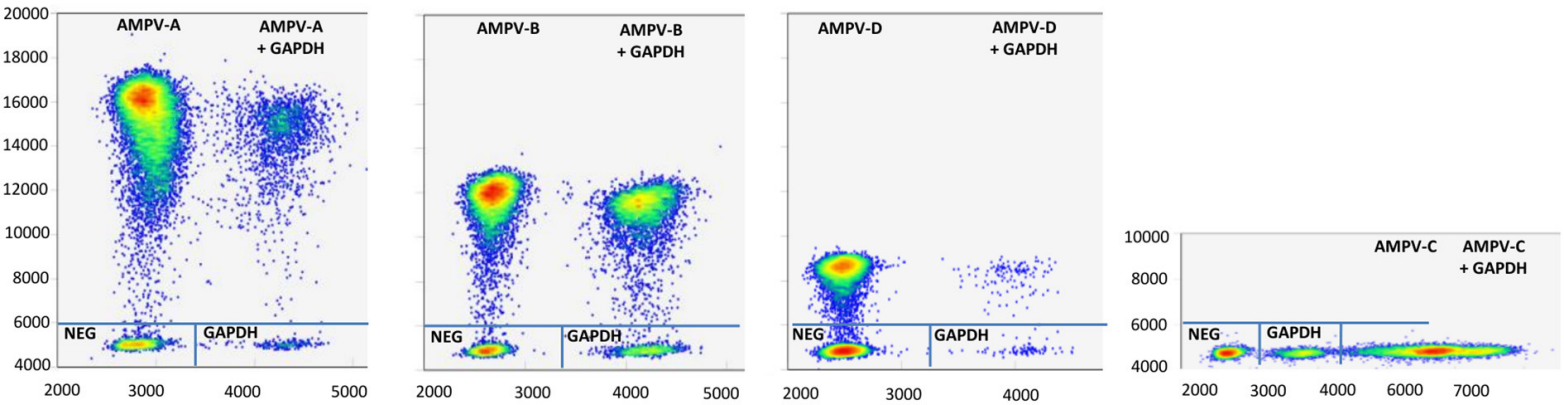
Graphical representation of the mean amplitude value for each cluster of the five-plex RT-ddPCR AMPV. Amplitude values in channel 1 (CH1, FAM) are represented on Y-axis. Amplitude values in channel 2 (CH2, HEX) are represented on X-axis. Clusters and the standard deviation of their respective amplitude are represented by dots and by lines on this dots, respectively. Statistical differences between AMPV-A, B, and D in CH1 and between AMPV-C and GAPDH in CH2 were determined by Krustall-Wallis tests (*p* < 0.0001).

The specificity of the five-plex RT-ddPCR assay was calculated to be 100% based on 31 AMPVs representing the four recognized subgroups of AMPV (Table 2). All the strains were correctly assigned to their specific subgroup based on their amplitude values. For CH1, a mean amplitude value of 15,312 was obtained for subgroup A (standard deviation (SD 677, *N* = 10), 11,681 for subgroup B (SD 777, *N* = 12), and 8,482 for subgroup D (SD 30, *N* = 2) (Supplementary Table S1). For CH2, mean amplitude values were 4,114 for GAPDH (SD 116, *N* = 23) and 5,649 for subgroup AMPV C's of both lineages (SD 171, *N* = 7) (Supplementary Table S1). Results of Kruskal-Wallis test showed significant differences between subgroup clusters (*p*-value <0.0001) for CH1 and CH2 amplitude (Figure 2). Probes were specific to their subgroup and did not cross-react. No droplet was observed in non-specific clusters. No AMPV-positive results were obtained from the 35 non-AMPV viruses, including the closely related HMPV and RSV (Table 2). Concerning the AMPV-positive tracheal swab samples, all were detected and assigned to their expected subgroup. Additionally, six tracheal swab samples from non-infected SPF birds (turkeys, ducks and chickens, two samples per species) were screened and were only GAPDH-positives (Table 4). All test samples were also positive for the GAPDH.

**Table 4:** Detection of AMPV in tracheal swabs from previous experimental trials.

**Virus**	**Species**		
	**Turkey**	**Duck**	**Chicken**
AMPV-A	5/5	/	5/5
AMPV-B	10/10	/	/
AMPV-C	2/2	3/3	/
AMPV-D	10/10	/	/
Negative	0/2	0/2	0/2

Results expression: positives number detected/total number screened); /, not available.

### Sensitivity and linearity

The estimation of the LoD was the same for the four subgroups and was equal to 10^1^ TCID_50_/ml, corresponding to one to three copies/μl of extracted viral RNA (Table 5).

**Table 5:** Limits of detection and quantification.

**Sub-group**	**Viral titer^a^**	**Assay 1^b^**			**Assay 2^b^**			**Copies/μl**	***R***
		**Rep 1**	**Rep 2**	**Rep 3**	**Rep 1**	**Rep 2**	**Mean +/– SD (CV)**		
A	4	3.27	3.25	3.39	3.23	3.21	3.27 +/**–** 0.07 (2.2)		0.9971
	3	2.32	2.28	2.30	2.25	2.18	2.27 +/**–** 0.05 (2.4)		
	2	1.26	1.28	1.25	1.16	1.25	1.24 +/**–** 0.05 (3.9)		
	1	0 **.46**	0 **.53**	0 **.51**	0 **.36**	0 **.32**	0**.44** ***±***/– 0**.09 (20.8)**	3	
	0	N	N	N	N	N			
B	4	2.83	2.87	2.89	2.87	2.89	2.87 +/**–** 0.03 (0.9)		0.9990
	3	1.81	1.80	1.81	1.84	1.92	1.84 +/**–** 0.05 (2.6)		
	2	0.88	0.89	0.94	0.94	0.91	0.91±/–0.03 (3.4)	8	
	**1**	**0**.**26**	**0**.**36**	**0**.**18**	**0**.**15**	**0**.**40**	**0**.**27 +/– 0**.**11 (41**.**4)**	**2**	
	0	N	N	N	N	N			
C	4	2.84	2.82	2.70	2.79	2.79	2.79 +/**–** 0.05 (1.9)		0.9999
	3	1.84	1.91	1.85	1.85	1.83	1.86 +/**–** 0.03 (1.7)		
	2	0.81	1.00	0.78	0.90	0.94	0.89±/–0.09 (10.3)	8	
	**1**	**−0**.**10**	**−0**.**22**	**−0**.**30**	**−0**.**10**	**−0**.**40**	**−0**.**22 +/−0**.**13 (58**.**7)**	**1**	
	0	N	N	N	N	N			
D	4	2.57	2.56	2.57	2.58	2.58	2.57 +/**–** 0.01 (0.3)		1.0000
	3	1.55	1.58	1.62	1.59	1.65	1.60 +/**–** 0.04 (2.3)		
	2	0.51	0.67	0.59	0.67	0.59	0.61±/–0.07 (11.5)	4	
	**1**	**−0**.**15**	**0**.**15**	**0**.**15**	**−0**.**15**	**0**.**04**	**0 +/– 0**.**15**	**1**	
	0	N	N	N	N	N			

a : Log_10_ (TCID_50_/ml).

b : Log_10_ (absolute copies/μl of extracted viral RNA), R^2^, Co-efficient of determination of the linear regression curve; Rep, replicate number; SD, standard deviation; CV, co-efficient of variation expressed in %; N, not detected; bold line: limit of detection; underlined line: limit of quantification.

For ddPCR, the number of target molecules per droplet is important for accurate calculation of the concentration of initial target molecules by Poisson correction statistics. A range from 10^4^ to 10^1^ TCID_50_/ml was selected to evaluate the linearity of the method. A 2D plot of viral titer (log_10_ TCID_50_/ml) against absolute quantification (log_10_ copies/μl) was generated for the four reference viruses. The *R*^2^ of the linear regression model showed a linear correlation from 10^4^ to 10^1^ TCID_50_/ml for subgroup A (*R*^2^ = 0.9971) and from 10^4^ to 10^2^ TCID_50_/ml for subgroups B, C and D (*R*^2^ = 0.9990, 0.9999, and 1, respectively) (Supplementary Figure S1). These data were correlated with the sensitivity results showing a decrease of precision when the mean number of positive droplets was very low (Table 3). The range between 10^2^ and 10^1^ TCID_50_/ml for subgroups B, C, and D was detectable but not quantifiable due to a high percentage of variation in the results.

## Discussion

Multiplex PCRs are difficult to develop mainly because of the competition between the molecules in the same reaction mixture, however, when one PCR reaction mixture containing many different molecules can be separated into thousands of individual reactions (droplets in the case of ddPCR), then competition is drastically reduced without losing the multiplexing capabilities of the reaction as a whole. Here, a five-plex RT-ddPCR for the detection and differentiation of any one of the four recognized AMPV subgroups has been achieved using an amplitude-based multiplexing approach with double-quenched probes at different final concentrations coupled with the same dye (31) to achieve sufficient cluster separation for clear identification of each subgroup. Amplitude reads on CH1 corresponding to FAM labeled probes were more extensive on the value scale than those of HEX, and for this reason, three targets (AMPV-A, B and D) were separated using this fluorochrome. Only two targets were used with HEX (AMPV-C and GAPDH). Ten clearly defined clusters were obtained: one for each AMPV subgroup (four in total), one for each AMPV subgroup in association with GAPDH (four in total), one for GAPDH only and a final cluster for negative droplets absent of target molecules (Figure 1).

Prior to the current study, all but one subgroup specific AMPV PCR have targeted open reading frames (ORFs) of high nucleotide variability between subgroups such as the SH or G ORFs (26, 28–30, 36) because sequences specific to each subgroup in these regions are in abundance. However, the problem with this approach is that hypervariable sequences are subject to frequent nucleotide change implicating potential mismatch with primers and/or probes, as has been previously evoked (25). The current RT-ddPCR has been therefore developed using a low-variability region in the L ORF (4, 34) located between nucleotide positions 5′ 1,980 and 5′ 2,158 to overcome the above-mentioned limitations. Other conserved ORFs in the AMPV genome, such as that coding for the nucleocapsid protein (*N*), which are more abundant as viral RNA molecules in an infected cell due to their proximity to the 3′ leader end of the genome (37, 38), were initially considered as targets for the design. However, no suitable regions could be found.

As described, the main focus of the current study was on the multiplexing aspect of the RT-ddPCR and not on its quantification capabilities. However, one advantage of developing ddPCRs is that absolute quantities of target molecules are calculated automatically without the need for the development of reference standards. Quantities in ddPCR are calculated using the “Poisson distribution model” based on the total number of droplets in a given reaction volume and the number of positive droplets within this population. This type of quantity estimation is not established by referring to a concentration curve generated using RNA transcripts that may give imprecise measures due to the initial method of dosage. Thus, quantities of target molecules in the initial sample as calculated by ddPCR could be interpreted to be more accurate; however, the efficiency of the RT-PCR also needs to be taken into account. The LoDs of the five-plex RT-ddPCR developed in the current study were in the same order as those described previously for subgroup specific AMPV real-time RT-PCR tests (26, 28). However, as described above, the differences in the methods of quantification by RT-ddPCR and real-time RT-PCR means that comparisons should be treated with caution.

In conclusion, an end-point RT-ddPCR has been developed that is capable of identifying AMPV-A, B, C and D in a single reaction. This multiplex RT-ddPCR is based on a highly conserved region of the genome and thus should have less risk of being affected by viral genome evolution and the impact it has on the specificity of such tests. A diagnostic test of such type with greater durability in terms of specificity will also help improve the accuracy when assessing the distribution of AMPV-A, B-C and D in the field. Further developments of this method could include the ability to detect co-infections with different AMPV wild type viruses or of wild type virus and vaccine strains, both unreported to date. Finally, because the primers used for this test also match the L gene sequences of the two newly identified gull and parakeet AMPVs, it may be possible to extend this five-plex RT-ddPCR to a seven-plex using machines offering more numerous fluorescent color options.

## Data availability statement

The raw data supporting the conclusions of this article will be made available by the authors, without undue reservation.

## Author contributions

EL: study design, experiments, analyses, and writing of the manuscript. SB: statistical analysis of data. CA: experiments. CC and NE: critical analysis and contribution to manuscript text. PB: supervision of experiments and manuscript text. All authors reviewed and approved the manuscript.

## Funding

This work was supported by the “Appel à Manifestation d'Intérêt Transversalité Inter-Laboratoires et autres Directions” of Anses, the Département des Côtes d'Armor, Saint-Brieuc Armor Agglomération, the Conseil Général des Côtes d'Armor (CG22), and the Conseil Régional de Bretagne.

## Conflict of interest

The authors declare that the research was conducted in the absence of any commercial or financial relationships that could be construed as a potential conflict of interest.

## Publisher's note

All claims expressed in this article are solely those of the authors and do not necessarily represent those of their affiliated organizations, or those of the publisher, the editors and the reviewers. Any product that may be evaluated in this article, or claim that may be made by its manufacturer, is not guaranteed or endorsed by the publisher.

## Supplementary material

Supplementary Figure S1 Dose-response of reference viruses for each tested AMPV subgroup (A, B, C, and D). Viral titer of each dilution of each reference virus is represented on X-axis (expressed in log_10_ TCID_50_/ml). Corresponding values obtained by the five-plex RT-ddPCR AMPV are represented on Y-axis (expressed in log_10_ copies/μl) The coefficient of determination (*R*^2^) of the linear regression model for each subgroup is indicated.

## Abbreviations

AMPV: avian metapneumoviruses
CH1: channel 1
CH2: channel 2
CV: co-efficient of variation
ddPCR: droplet digital PCR
FAM: 6-carboxyfluorescein
GAPDH: Glyceraldehyde-3-phosphate dehydrogenase
HEX: hexachloro-fluorescein phosphoramidite
HMPV: human metapneumovirus
LoD: limit of detection
LoQ: limit of quantification
ORF: open reading frame
*R* ^2^: co-efficient of determination
RSV: respiratory syncytial virus
SD: standard deviation
SPF: specific pathogen-free.

## References

[1] Maes P Amarasinghe GK Ayllon MA Basler CF Bavari S Blasdell KR. Taxonomy of the order mononegavirales: second update 2018. Arch Virol. (2019) 164:1233–44. 10.1007/s00705-018-04126-4 30663023 PMC6460460

[2] Rautenschlein S. Avian metapneumovirus. In: Swayne DE, editor. Diseases of Poultry. 1. 14 ed. Hoboken, NJ: John Wiley & Sons (2019). p. 135–43, 60–66.

[3] Bäyon-Auboyer MH Jestin V Toquin D Cherbonnel M Eterradossi N. Comparison of F-, G- and N-based RT-PCR protocols with conventional virological procedures for the detection and typing of turkey rhinotracheitis virus. Arch Virol. (1999) 144:1091–109. 10.1007/s007050050572 10446646

[4] Brown PA Lemaitre E Briand FX Courtillon C Guionie O Allee C. Molecular comparisons of full length metapneumovirus (MPV) genomes, including newly determined French AMPV-C and -D isolates, further supports possible subclassification within the MPV genus. PLoS ONE. (2014) 9:e102740. 10.1371/journal.pone.0102740 25036224 PMC4103871

[5] Eterradossi N Toquin D Guittet M Bennejean G. Evaluation of different turkey rhinotracheitis viruses used as antigens for serological testing following live vaccination and challenge. J Vet Med Series B. (1995) 42:175–86. 10.1111/j.1439-0450.1995.tb00698.x 8553711

[6] Juhasz K Easton AJ. Extensive sequence variation in the attachment (G) protein gene of avian pneumovirus: evidence for two distinct subgroups. J Gen Virol. (1994) 75:2873–80. 10.1099/0022-1317-75-11-2873 7964599

[7] Seal BS. Matrix protein gene nucleotide and predicted amino acid sequence demonstrate that the first US avian pneumovirus isolate is distinct from European strains. Virus Res. (1998) 58:45–52. 10.1016/S0168-1702(98)00098-7 9879761

[8] Toquin D Guionie O Jestin V Zwingelstein F Allee C Eterradossi N. European and American subgroup C isolates of avian metapneumovirus belong to different genetic lineages. Virus Genes. (2006) 32:97–103. 10.1007/s11262-005-5850-3 16525740

[9] Retallack H Clubb S DeRisi JL. Genome sequence of a divergent avian metapneumovirus from a monk parakeet (*Myiopsitta monachus*). Microbiol Resour Announc. (2019) 8:e00284–19. 10.1128/MRA.00284-19 31000555 PMC6473149

[10] Canuti M Kroyer ANK Ojkic D Whitney HG Robertson GJ Lang AS. Discovery and characterization of novel RNA viruses in aquatic north American wild birds. Viruses. (2019) 11:768. 10.3390/v11090768 31438486 PMC6784231

[11] Lee E Song MS Shin JY Lee YM Kim CJ Lee YS. Genetic characterization of avian metapneumovirus subtype C isolated from pheasants in a live bird market. Virus Res. (2007) 128:18–25. 10.1016/j.virusres.2007.03.029 17485129

[12] Picault JP Giraud P Drouin P Lamande J Toquin D Guegen C. Le syndrome de gonflement de la tête chez la poule et la pintade: Une étiologie commune avec la rhinotrachéite infectieuse de la dinde. L'Aviculteur. (1987) 481:74–5.

[13] Jardine CM Parmley EJ Buchanan T Nituch L Ojkic D. Avian metapneumovirus subtype C in wild waterfowl in Ontario, Canada. Transbound Emerg Dis. (2018) 65:1098–102. 10.1111/tbed.12832 29457370

[14] Naylor C Shaw K Britton P Cavanagh D. Appearance of type B avian Pneumovirus in great Britain. Avian Pathol. (1997) 26:327–38. 10.1080/03079459708419215 18483910

[15] Bäyon-Auboyer MH Arnauld C Toquin D Eterradossi N. Nucleotide sequences of the F, L and G protein genes of two non-A/non-B avian pneumoviruses (APV) reveal a novel APV subgroup. J Gen Virol. (2000) 81(Pt 11):2723–33. 10.1099/0022-1317-81-11-2723 11038385

[16] Giraud P Bennejean G Guittet M Toquin D. Turkey rhinotracheitis in France: preliminary investigations on a ciliostatic virus. Vet Rec. (1986) 119:606–7. 3027949

[17] Senne DA Edson Pederson JC Panigrahy B. Avian Pneumovirus Update. Schaumburg, IL: American Veterinary Medical Association (1997).

[18] Toquin D Bayon-Auboyer MH Eterradossi N Jestin V. Isolation of a pneumovirus from a Muscovy duck. Vet Rec. (1999) 23:680. 25705778

[19] Shin HJ Nagaraja KV McComb B Halvorson DA Jirjis FF Shaw DP. Isolation of avian pneumovirus from mallard ducks that is genetically similar to viruses isolated from neighboring commercial turkeys. Virus Res. (2002) 83:207–12. 10.1016/S0168-1702(01)00402-6 11864753

[20] Cook JK. Avian pneumovirus infections of turkeys and chickens. Vet J. (2000) 160:118–25. 10.1053/tvjl.2000.0486 10985803

[21] Jones RC Naylor CJ Bradbury JM Savage CE Worthington K Williams RA. Isolation of a turkey rhinotracheitis-like virus from broiler breeder chickens in England. Vet Rec. (1991) 129:509–10. 1664552

[22] Mase M Yamaguchi S Tsukamoto K Imada T Imai K Nakamura K. Presence of avian pneumovirus subtypes A and B in Japan. Avian Dis. (2003) 47:481–4. 10.1637/0005-2086(2003)0470481:POAPSA 2.0.CO;2 12887210

[23] Picault J-P Giraud P Drouin P Guittet M Bennejean G Lamandé J. Isolation of a TRTV-like virus from chickens with swollen-head syndrome. Vet Rec. (1987) 121:135. 10.1136/vr.121.6.135-a 3118557

[24] Wei L Zhu S Yan X Wang J Zhang C Liu S. Avian metapneumovirus subgroup C infection in chickens, China. Emerg Infect Dis. (2013) 19:1092–4. 10.3201/eid1907.121126 23763901 PMC3903454

[25] Brown PA Allée C Courtillon C Szerman N Lemaitre E Toquin D. Host specificity of avian metapneumoviruses. Avian Pathol. (2019) 48:311–8. 10.1080/03079457.2019.1584390 30777452

[26] Guionie O Toquin D Sellal E Bouley S Zwingelstein F Allee C. Laboratory evaluation of a quantitative real-time reverse transcription PCR assay for the detection and identification of the four subgroups of avian metapneumovirus. J Virol Methods. (2007) 139:150–8. 10.1016/j.jviromet.2006.09.022 17126416

[27] Lemaitre E Allee C Vabret A Eterradossi N Brown PA. Single reaction, real time RT-PCR detection of all known avian and human metapneumoviruses. J Virol Methods. (2018) 251:61–8. 10.1016/j.jviromet.2017.10.010 29030071 PMC7119483

[28] Cecchinato M Lupini C Munoz Pogoreltseva OS Listorti V Mondin A Drigo M. Development of a real-time RT-PCR assay for the simultaneous identification, quantitation and differentiation of avian metapneumovirus subtypes A and B. Avian Pathol. (2013) 42:283–9. 10.1080/03079457.2013.788130 23650927

[29] Kwon JS Lee HJ Jeong SH Park JY Hong YH Lee YJ. Isolation and characterization of avian metapneumovirus from chickens in Korea. J Vet Sci. (2010) 11:59–66. 10.4142/jvs.2010.11.1.59 20195066 PMC2833431

[30] Tucciarone CM Franzo G Legnardi M Pasotto D Lupini C Catelli E. Molecular survey on A, B, C and new avian metapneumovirus (aMPV) subtypes in wild birds of northern-central Italy. Vet Sci. (2022) 9. 10.3390/vetsci9070373 35878390 PMC9319881

[31] Whale AS Huggett JF Tzonev S. Fundamentals of multiplexing with digital PCR. Biomol Detect Quantif. (2016) 10:15–23. 10.1016/j.bdq.2016.05.002 27990345 PMC5154634

[32] Giraud P Le Gros FX Bouquet JF Toquin D Bennejean G. Turkey rhinotracheitis: isolation of a viral agent and first trials with experimental inactivated or attenuated vaccines. In: 36th Western Poultry Disease Conference. Davis, CA (1987).

[33] Reed LJ Muench H. A simple method of estimating fifty per cent endpoints. Am J Epidemiol. (1938) 27:493–7. 10.1093/oxfordjournals.aje.a118408

[34] Poch O Blumberg BM Bougueleret L Tordo N. Sequence comparison of five polymerases (L proteins) of unsegmented negative-strand RNA viruses: theoretical assignment of functional domains. J Gen Virol. (1990) 71:1153–62. 10.1099/0022-1317-71-5-1153 2161049

[35] Dobnik D Stebih D Blejec A Morisset D Zel J. Multiplex quantification of four DNA targets in one reaction with Bio-Rad droplet digital PCR system for GMO detection. Sci Rep. (2016) 6:35451. 10.1038/srep35451 27739510 PMC5064307

[36] Franzo G Drigo M Lupini C Catelli E Laconi A Listorti V. A sensitive, reproducible, and economic real-time reverse transcription PCR detecting avian metapneumovirus subtypes A and B. Avian Dis. (2014) 58:216–22. 10.1637/10676-092413-Reg.1 25055624

[37] Easton AJ Domachowske JB Rosenberg HF. Animal pneumoviruses: molecular genetics and pathogenesis. Clin Microbiol Rev. (2004) 17:390–412. 10.1128/CMR.17.2.390-412.2004 15084507 PMC387412

[38] Krempl C Murphy BR Collins PL. Recombinant respiratory syncytial virus with the G and F genes shifted to the promoter-proximal positions. J Virol. (2002) 76:11931–42. 10.1128/JVI.76.23.11931-11942.2002 12414935 PMC136893

