# Supplementary figures and images for: Avian metapneumovirus: A five-plex digital droplet RT-PCR method for identification of subgroups A, B, C, and D
Source: Front Vet Sci. 2022 Nov 15;9:1058294. doi: 10.3389/fvets.2022.1058294 (PMC9705331; doi:10.3389/fvets.2022.1058294)

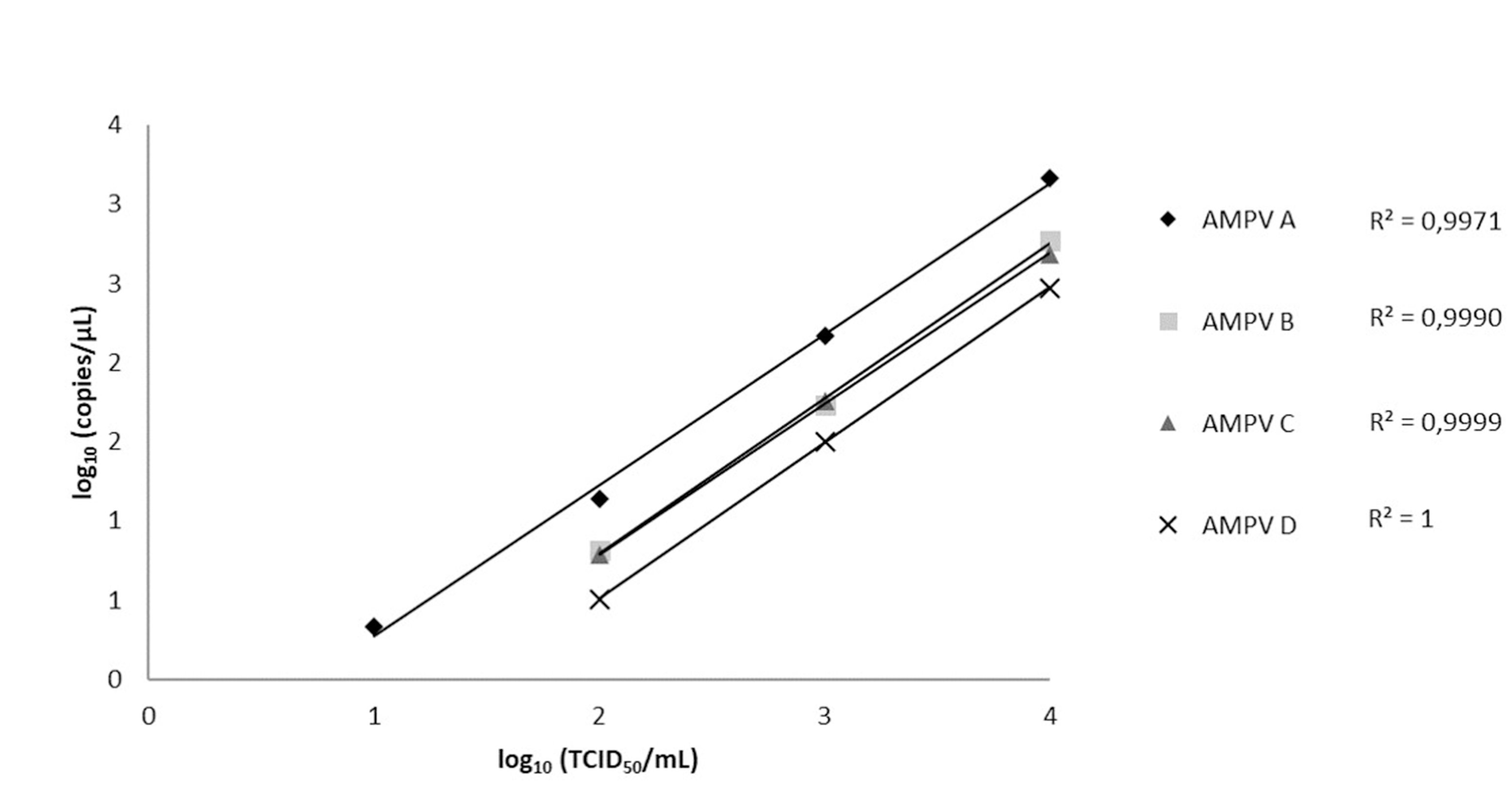

Supplement: Supplementary Figure S1 — Dose-response of reference viruses for each tested AMPV subgroup (A, B, C, and D). Viral titer of each dilution of each reference virus is represented on X-axis (expressed in log10 TCID50/ml). Corresponding values obtained by the five-plex RT-ddPCR AMPV are represented on Y-axis (expressed in log10 copies/μl) The coefficient of determination (R2) of the linear regression model for each subgroup is indicated. [file Image_1.TIFF]
