# Supplementary material for: Avian metapneumovirus: A five-plex digital droplet RT-PCR method for identification of subgroups A, B, C, and D
Source: Front Vet Sci. 2022 Nov 15;9:1058294. doi: 10.3389/fvets.2022.1058294 (PMC9705331; doi:10.3389/fvets.2022.1058294)
Supplement: Supplementary file 2 [file Table_1.docx]

**Supplementary Table 1: Amplitude values of clusters obtained for the specificity tests using 31 AMPV strains representing AMPV subgroups A, B, C and D in each of the channels.**

| Anses reference | Source | Subgroup | Species | CH1 Amplitude | | CH2 Amplitude | |
| --- | --- | --- | --- | --- | --- | --- | --- |
|  |  |  |  | Positive | Negative | Positive | Negative |
| TRT Nobilis | UK | **A** | Vaccine | 14787 | 5055 | 4430 | 2825 |
| Turkadin | UK | **A** | Vaccine | 14506 | 4941 | 4106 | 2735 |
| 85051 | France | **A** | Turkeys | 15562 | 4854 | 3929 | 2598 |
| 93084 | Israël | **A** | Ind | 14923 | 4880 | 4123 | 2670 |
| STG761/88 | Germany | **A** | Ind | 14810 | 4824 | 4116 | 2634 |
| STG854/88 | Germany | **A** | Ind | 15061 | 4878 | 4137 | 2612 |
| STGIII/88 | Germany | **A** | Ind | 14974 | 5050 | 4237 | 2911 |
| 3BOC18 | UK | **A** | Turkeys | 16175 | 5458 | 4194 | 2835 |
| 14/86/2 | UK | **A** | Turkeys | 16595 | 5194 | 4107 | 2675 |
| TRT 1125/91 | Germany | **A** | Ind | 15726 | 5062 | 4095 | 2568 |
|  |  | Mean AMPV A | | 15312 |  |  |  |
| Aviffa RTI | France | **B** | Vaccine | 11631 | 5245 | 4150 | 2870 |
| 86004 | France | **B** | Turkeys | 9948 | 5014 | 4034 | 2714 |
| 98103 | France | **B** | Turkeys | 11576 | 4903 | 4157 | 2746 |
| 85231 | France | **B** | Turkeys | 11568 | 5046 | 4074 | 2787 |
| 85234 | France | **B** | Turkeys | 12557 | 4987 | 4057 | 2659 |
| 86016 | France | **B** | Chickens | 11216 | 4951 | 3983 | 2751 |
| 86019 | France | **B** | Turkeys | 11428 | 4918 | 4165 | 2683 |
| 95021 | France | **B** | Turkeys | 11276 | 4852 | 4231 | 2738 |
| 96252 | France | **B** | Turkeys | 12766 | 5081 | 3935 | 2737 |
| 97104 | France | **B** | Turkeys | 12453 | 4966 | 3961 | 2649 |
| 98238 | France | **B** | Chickens | 12402 | 5027 | *3395* | *2631* |
| 00185 | France | **B** | Turkeys | 11354 | 5054 | 4269 | 2829 |
|  |  | Mean AMPV B | | 11681 |  |  |  |
| 99178 | France | **C** | Ducks |  | 4830 | 5580 | 2549 |
| 99214 | France | **C** | Ducks |  | 4873 | 5659 | 2733 |
| 99350 | France | **C** | Ducks |  | 5025 | 5591 | 2593 |
| 00094 | France | **C** | Ducks |  | 4958 | 5594 | 2690 |
| 04268 | France | **C** | Ducks |  | 4900 | 5504 | 3306 |
| 193ADV9802 | USA | **C** | Turkeys |  | 4770 | 5592 | 2602 |
| AV247/97 | USA | **C** | Turkeys |  | 5531 | 6024 | 3167 |
|  |  | Mean AMPV C | |  |  | 5649 |  |
| 85035 | France | **D** | Turkeys | 8503 | 5009 | 4127 | 2591 |
| 85053 | France | **D** | Turkeys | 8461 | 4885 | 4001 | 2492 |
|  |  | Mean AMPV D | | 8482 |  |  |  |

CH1: channel 1 (FAM) ; CH2: channel 2 (HEX)
